# Deep learning-based prediction of in-hospital mortality for sepsis

**DOI:** 10.1038/s41598-023-49890-9

**Published:** 2024-01-03

**Authors:** Li Yong, Liu Zhenzhou

**Affiliations:** College of Computer Science and Engineering, Northwest Normal University, Lanzhou, 730070 People’s Republic of China

**Keywords:** Experimental models of disease, Computer science

## Abstract

As a serious blood infection disease, sepsis is characterized by a high mortality risk and many complications. Accurate assessment of mortality risk of patients with sepsis can help physicians in Intensive Care Unit make optimal clinical decisions, which in turn can effectively save patients’ lives. However, most of the current clinical models used for assessing mortality risk in sepsis patients are based on conventional indicators. Unfortunately, some of the conventional indicators have been shown to be inapplicable in the accurate clinical diagnosis nowadays. Meanwhile, traditional evaluation models only focus on a small amount of personal data, causing misdiagnosis of sepsis patients. We refine the core indicators for mortality risk assessment of sepsis from massive clinical electronic medical records with machine learning, and propose a new mortality risk assessment model, DGFSD, for sepsis patients based on deep learning. The DGFSD model can not only learn individual clinical information about unassessed patients, but also obtain information about the structure of the similarity graph between diagnosed patients and patients to be assessed. Numerous experiments have shown that the accuracy of the DGFSD model is superior to baseline methods, and can significantly improve the efficiency of clinical auxiliary diagnosis.

## Introduction

Sepsis is an acute systemic infection that occurs when various pathogenic bacteria invade the circulation and produce toxins. As a serious blood infection disease, sepsis is characterized by a high mortality risk and many complications^1,2^. World Health Organization (WHO) shows that in 2017, about 11 million patients with sepsis worldwide were at risk of death. Severe sepsis can lead to multiple organ failure in patients, with a mortality rate of 9.7%^3,4^. Especially in patients who develop septic shock, the mortality rate can reach more than 40%^5^. In 2018, sepsis was responsible for 15% of all neonatal deaths worldwide. In addition, according to the WHO’s Executive Board, sepsis leads to an economic burden of more than $24 billion per year, representing 6.2% of total hospital costs. In recent years, despite some advances have been witnessed in management and treatment, sepsis diagnosis and treatment continues to be a focal area of research in global health^6–8^. Early identification of septic patients at high risk of death during patient care has been shown to be effective in improving patient outcomes^9–11^.

However, there are still some challenges in the current risk assessment methods of mortality in sepsis patients. First of all, the indicators recognized in the current widespread clinical scoring methodology are based on the empirical findings of traditional medical experts, and some of the conventional indicators have been shown to be inapplicable in the accurate clinical diagnosis nowadays^10^. What’s more, the rapid onset of sepsis results in the inability of septic patients to accumulate as much individual clinical records as patients with chronic illnesses, making it challenging to predict the risk of death in septic patients. Last but not least, the accuracy of existing clinical scoring methods such as SOFA and SAPS II is deficiency, resulting in ineffective assessment of mortality risk in sepsis patients compared to machine learning^12^.

In recent years, a large number of researchers have been committed to addressing challenges of mortality risk assessment for sepsis. By using statistical methods, they identified core indicators for assessing mortality risk in patients with sepsis. Using Cox regression model and subgroup analysis, Wang et al.^13^ identified the ratio of blood urea nitrogen to serum albumin as an important predictor of death in sepsis patients. Dias et al.^14^ found that afebrile patients with sepsis admitted to the ICU from the ward had higher mortality than febrile patients by using multivariate analysis. Hu et al.^15^ used Cox risk model and multifactorial regression analysis to demonstrate that although albumin level is one of the indicators for assessing disease severity in patients with sepsis, hypoproteinemia has no significant effect on the risk of death in patients with sepsis. Risk assessment of mortality in septic patients is generally based on medical datasets and machine learning models for analytical prediction^16,17^. Hou et al.^18^ used the XGboost model to predict 30-day mortality risk in septic patients in the ICU, demonstrating the clinically significant predictive value of XGboost. On the other hand, Kong et al.^10^ compared the predictive performance of four machine learning methods and SAPS II., and showed that the GBM, LASSO, and linear regression (LNR) models had excellent scalability, whereas the random forests(RF) model underestimated high-risk septic patients, and SAPS II is slightly negative. Perng et al.^19^ has convinced himself through extensive experiments that convolutional neural networks(CNN) with softmax model outperforms autoencoder, principal component analysis(PCA), and machine learning such as K-nearest neighbor(KNN), support vector machines(SVM), and RF.

Despite the fact that existing studies have yielded some results, there are still some drawbacks. In terms of obtaining core indicators for the assessment of risk of mortality for sepsis, the number of indicators that can be identified by statistical methods is scarce. In addition, although statistical methods can determine that certain indicators have a significant impact on the risk of death in patients with sepsis, statistical methods cannot rank the impact of multiple highly significant indicators on the mortality of sepsis patients, in order to further determine which indicators are the most core indicators. With respect to mortality risk assessment in septic patients, machine learning still focus only on individual clinical records of septic patients with limited records, restricting assessment accuracy.

In this paper, we extract core indicators for mortality risk assessment of sepsis with machine learning, and propose a new mortality risk assessment model, DGFSD, for sepsis patients based on deep learning. Above all, we use machine learning model, XGboost, and adopt the recommendations of clinical experts to filter out the core indicators required for risk assessment, based on a massive amount of internationally available EMRs of sepsis patients. Then, a similarity connectivity graph of sepsis patients is constructed by patients similarity graph to connect patients with similar indicators. After that, we propose a mortality risk prediction model DGFSD for sepsis patients by constructing deep neural network (DNN) and graph convolutional network (GCN). The DGFSD model can not only learn individual clinical information about unassessed patients, but also obtain information about the structure of the similarity graph between diagnosed patients and patients to be assessed. Finally, we perform multiple experiments of the DGFSD model on MIMIC-III, an internationally recognized open medical dataset, and compare the performance of DGFSD with other classical machine learning. The experimental results show the superiority of the DGFSD model in predicting the risk of death of sepsis, and the DGFSD model can reach the criteria for clinical auxiliary diagnostic of sepsis.

## Method

### Dataset

MIMIC-III^20^ is a large-scale public dataset jointly released by the Computational Physiology Laboratory of Massachusetts Institute of Technology, the Beth Israel Medical Center, and Philips Medical. ICU patients records from 2001 to 2012 at Beth Israel Deaconess Medical Center were collected in MIMIC-III, which includes data from many types of ICUs, such as the Surgical Care Unit, Medical Care Unit, and Trauma Surgical Care Unit. MIMIC-III contains patient’s vital signs trend data and patient’s clinical data, it is divided into four major categories: patient’s basic information as well as transfer information category, patient's hospital outpatient related information category, patient’s ICU related information category, and auxiliary information category, according to the degree of relevance of the record content. The four categories include 26 data tables such as hospitalization table, discharge table, date-type schedule, medical staff table, and monitoring situation table. etc. Researchers must pass tests in order to gain approval from the manager to use the dataset. We were approved to extract data from the MIMIC-III for research purposes after testing through the Citi Program.

### Clinical Characteristics

We followed the criteria below to pick out patients: (1) Patients older than 18 years of age; (2) Patients diagnosed with sepsis according to the third international consensus definition of sepsis and septic shock; (3) Analyze each admission of sepsis patients as an independent sample.

A total of 9432 patients with sepsis were included in the study. 1926 patients died, about 20.4% of the total. According to research by Hu et al.^12^, we employed international standardized ratios (INR) and so on as indicators for constructing a mortality risk prediction model by referring to established scoring tools such as SAPS II and APACHE III. The indicators contained both laboratory indicators and vital signs. Laboratory indicators included maximum values of serum creatinine, anion gap, lactate, blood urinary nitrogen (BUN), PH, white blood cell, bicarbonate, ionized calcium, serum calcium, serum chloride, serum sodium, serum potassium, blood glucose, INR, prothrombin time (PT), partial thromboplastin time (PTT), alanine aminotransferase (ALT), alkaline phosphatase (ALP), aspartate aminotransferase (AST), total bilirubin, creatine kinase MB, and lactate dehydrogenase, as well as minimum values of hematocrit and albumin. Vital signs included age, mean values of heart rate, respiratory rate, and body temperature, minimum values of oxygen saturation and the Glasgow Coma Scale (GCS) score (Supplementary Table S1).

We excluded indicators of septic patients with more than 30% missing values to generate a usable dataset (Supplementary Figure S1), and divided the training and test sets in a 7:3 ratio. Furthermore, we interpolated the missing values using the reference values of the indicators^21^.

### Baseline

The goal of mortality risk assessment in septic patients is to accurately categorize patient outcomes, so it is essentially a binary classification task. For this reason, we choose accuracy (ACC) as an evaluation metric to assess the performance of the models. Considering that the dataset is unbalanced, we use SMOTE^22^ algorithm to oversample the dataset, so that the utility of models can be reflected actually by the accuracy. ACC can be described as follows:

1 $$ACC=\frac{{S}_{r}+{D}_{r}}{{S}_{r}+{D}_{r}+{S}_{f}+{D}_{f}}$$

where ${S}_{r}$ denotes the number of actual surviving sepsis patients judged to be alive by the model, ${D}_{r}$ is the number of sepsis patients whose actual deaths were determined as deaths by the model. ${S}_{f}$ represents the number of patients who are judged as dead by the model based on the actual survival of sepsis patients. ${D}_{f}$ is used as the number of patients with sepsis that the model determines as surviving when they actually die.

To verify the effectiveness of the DGFSD model, we compared the DGFSD model with the following baseline models:

- Decision tree classification (DT): DT employs a tree structure and uses hierarchical reasoning to achieve the final classification. A decision tree is generally represented by a root node, internal nodes and leaf nodes. We define the root node as the full sample of septic patients, the internal nodes as the septic patient feature attribute, and the leaf nodes represent the final decision result of the patient. For prediction, judgment is made inside the tree with eigenvalues, and based on the judgment, it decides which branch node to enter until it reaches the leaf node to get the classification result.
- KNN: KNN predicts new data points by searching for the K most similar instances in the entire dataset and summarizing their output variables. We used KNN to make predictions about in-hospital outcomes for a particular septic patient by searching for information about similar patients.
- Logistic regression (LR): LR is mainly used to solve binary classification problems. LR calculates the probability of occurrence of a patient's outcome by accepting information about the characteristics of the sepsis patient's data. In particular, LR outperforms clinical scoring methods^12^.

Moreover, we perform ablation experiments by eliminating some of the modules in the DGFSD model, and the comparison methods involved include:

- DGFSD-D-LR: Information about the structure of the similarity graph between patients is not considered, only obtain information about the individual clinical information of the patients.
- DGFSD-G: Information about the individual clinical information of the septic patients is not considered, only obtain information about the structure of the similarity graph between patients.

### Problem definition

The clinical records of patients with sepsis can be represented as $S\in {R}^{n*d}$, where $S=\{{s}_{1},{s}_{2},{s}_{3},\dots ,{s}_{n}\}$ and ${S}_{i}$ denotes the *i*th sample, $d=\{albumin,alp,alt,\dots ,age\}$ indicates the physical indicators, such as albumin, AST, and age. etc. In order to compensate for the issue of insufficient individual clinical data of septic patients, we obtained a large amount of patient information with similar indicators to individual patients by similarity calculation, denoted as similarity matrix $A\in {R}^{n*n}$.

The problem of assessing the risk of death in septic patients can be formalized as follows: given individual clinical data $S$ on septic patients and information on similar patients $A$, the decision objective is to calculate the probability of the risk of death in septic patients through the DGFSD model ${P}_{h}=DGFSD\{S,A\}$.

### Model description

**Figure 1:**
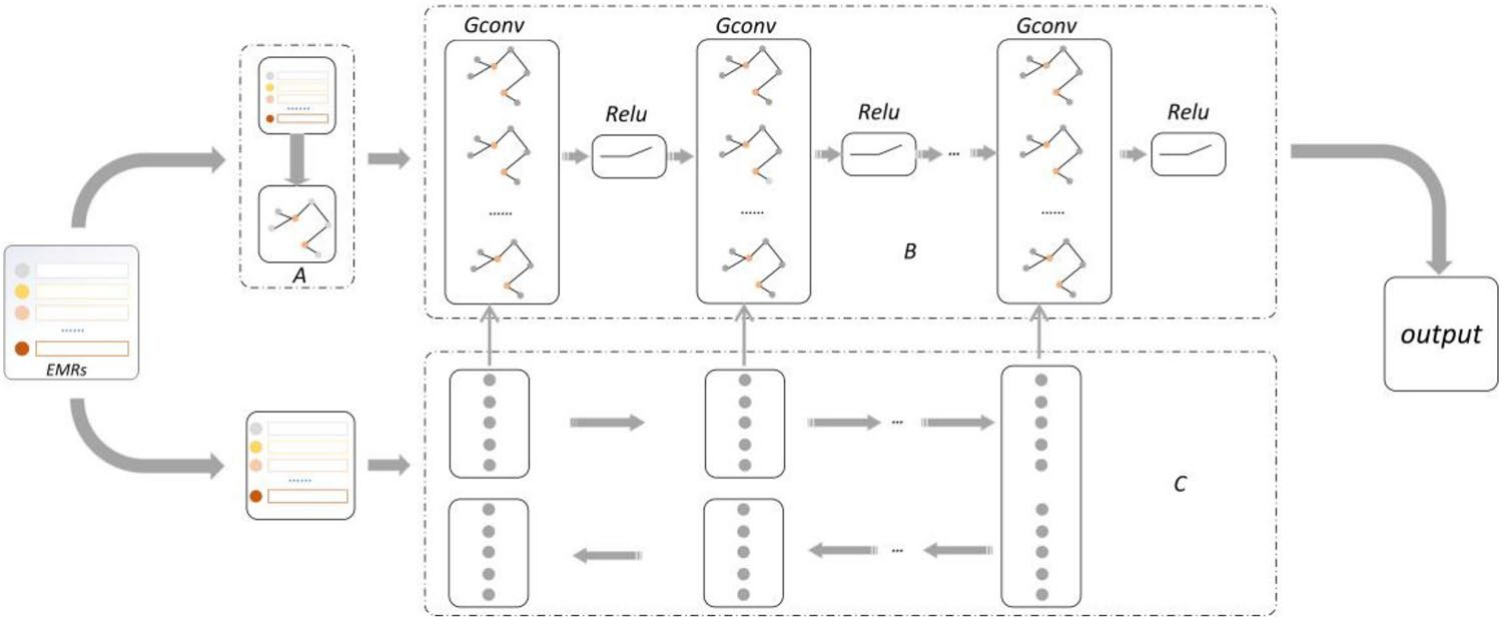
DGFSD, a mortality risk assessment model for sepsis patients based on deep learning. (**A**) represents the construction of a patients similarity graph between similar sepsis patients by similarity formula; (**B**) denotes the GCN module and the DGFSD model learns graph structure information between evaluated and unevaluated patients via the GCN module; (**C**) represents the DNN module, through which DGFSD learns data information from unassessed patients. After integrating the information learned by the DNN into the GCN, the DGFSD model is able to learn two representations of the information, resulting in a more accurate assessment of the risk of death.

To more accurately assess the risk of mortality in patients with sepsis, we first construct a patients similarity graph based on the original sepsis patient records. Then we input the patients’ data and patients similarity graph into autoencoder and GCN, respectively. The DGFSD model connects each layer of the autoencoder to the corresponding GCN layer so that a representation specific to the autoencoder can be integrated into a structurally aware representation of the GCN, and finally output the prediction result through GCN (Fig. 1).

### Patients similarity graph

For each septic patient, we locate the top-k similar patients and set up edges to connect them. The formula for calculating the similarity between patients $i$ and $j$ can be described as:

2 $${X}_{i,j}={e}^{-\frac{{|\left|{S}_{i}-{S}_{j}\right||}^{2}}{2}}$$

By calculating the similarity matrix $X$, we select the top-k similarities of each patient and construct an undirected patients similarity graph. Finally, the patient's adjacency matrix $A$ can be obtained from the non-graph data of the septic patient.

### DNN module

Variations of the basic autoencoder include masked autoencoders, convolutional autoencoder, LSTM encoder-decoder, adversarial autoencoder and deep autoencoder. etc^23–27^. We opted for the basic autoencoder to learn the clinical data representation of septic patients. We suppose there are $L$ layers in the autoencoder and $l$ denotes the number of layers. Then the septic patient clinical data representation ${H}^{(l)}$ learned by the $l$ th layer encoder can be represented as follows:

3 $${H}^{(l)}=\varnothing [{{W}_{e}^{(l)}H}^{\left(l-1\right)}+{b}_{e}^{\left(l\right)}]$$

where $\varnothing$ is the activation function of the fully connected layer, ${W}_{e}^{(l)}$ and ${b}_{e}^{(l)}$ denote the weight matrix and bias of the $l$ th layer of the encoder, respectively. In addition, the input to 0th layer of the encoder is septic patient clinical records $S$.

The decoder section reconstructs the input data by the following description:

4 $${H}^{(l)}=\varnothing [{W}_{d}^{\left(l\right)}{H}^{\left(l-1\right)}+{b}_{d}^{(l)}]$$

where ${W}_{d}^{(l)}$ and ${b}_{d}^{(l)}$ are the weight matrix and bias of the lth layer of the decoder, respectively.

### GCN module

We enable the GCN to learn both kinds of information by integrating the clinical data representation learned by the DNN into the GCN. The representation ${G}^{(l)}$, learned by the GCN module at $l$ th layer, can be described as follows:

5 $${G}^{(l)}=\varnothing [{\widetilde{D}}^{-\frac{1}{2}}\widetilde{A}{\widetilde{D}}^{-\frac{1}{2}}{G}^{(l-1)}{W}^{(l-1)}]$$

6 $$\widetilde{A}=A+I$$

7 $${\widetilde{D}}_{ii}={\sum }_{j}{\widetilde{A}}_{ij}$$

where $W$ is the weight matrix, $I$ is the unit diagonal matrix of the adjacency matrix $A$. In order to combine the individual information of septic patients learned from the autoencoder into the GCN, we merged ${H}^{(l-1)}$ with ${G}^{(l-1)}$ in the following way:

8 $${\widetilde{G}}^{(l-1)}=\left(1-\epsilon \right){G}^{\left(l-1\right)}+\epsilon {H}^{(l-1)}$$

where ϵ is an equilibrium coefficient. Due to reduce hyperparameter search in DGFSD, ϵ is set to 0.5, making the representation of GCN module and DNN module equally important^28^.We combine the autoencoder and the GCN layer by layer through Eq. (8) and use ${\widetilde{G}}^{(l-1)}$ as the input to the $l$ th layer in the GCN. At this point, the new data representation is as follows:

9 $${G}^{(l)}=\varnothing [{\widetilde{D}}^{-\frac{1}{2}}\widetilde{A}{\widetilde{D}}^{-\frac{1}{2}}{\widetilde{G}}^{(l-1)}{W}^{(l-1)}]$$

From Eq. (9), it can be seen that the individual clinical information of septic patients learned by the autoencoder ${H}^{\left(l-1\right)}$ will be propagated in the GCN through the normalized adjacency matrix ${\widetilde{D}}^{-\frac{1}{2}}\widetilde{A}{\widetilde{D}}^{-\frac{1}{2}}$.

As the beginning of the GCN layer, we input the individual data $S$ of the septic patient into the first GCN layer, at which point the first GCN layer is represented as shown below:

10 $${G}^{(1)}=\varnothing [\widetilde{A}{\widetilde{D}}^{-\frac{1}{2}}S{W}^{\left(1\right)}]$$

The final layer of the GCN module is a binary classification layer with Relu functionality, the final representation of which is shown below:

11 $$G=Relu[{\widetilde{D}}^{-\frac{1}{2}}\widetilde{A}{\widetilde{D}}^{-\frac{1}{2}}{Z}^{(l)}{W}^{(l)}]$$

The result ${g}_{i,j}\in G$ indicates that septic patient $i$ was assessed for outcome $j$, at which point $G$ is considered as a probability distribution.

## Results

We evaluate the DGFSD model based on records from a large number of septic patients in MIMIC-III and compare the results of the DGFSD model with baseline models. Through extensive experimental comparison and analysis, we have obtained the following three main conclusions.

- **RC1**. The results of our experiments indicate that serum sodium, serum potassium, and BUN are not central to the assessment of mortality risk in patients with sepsis in the accurate clinical diagnosis.
- **RC2. **We compare DGFSD with baseline models and find that the DGFSD model is more prominent than baseline models, indicating that the DGFSD model can be effectively applied in clinical auxiliary diagnosis.
- **RC3. **The DGFSD model can not only learn individual clinical information of undiagnosed sepsis patients, but also obtain similarity graph structure information between diagnosed and undiagnosed patients, thereby improving the evaluation accuracy of the model.

### Core indicators analysis (RC1)

**Figure 2:**
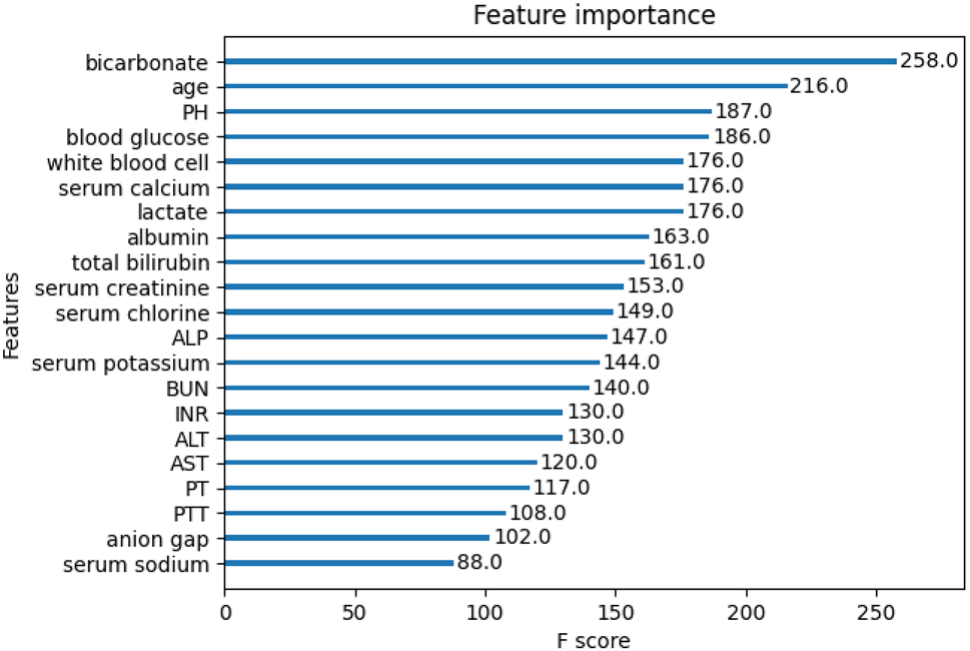
Ranking of baseline indicators’ importance.

The baseline indicators we used are shown in Table 1. We first balance the dataset using SMOTE algorithm and then use the XGboost to obtain the importance ranks of the baseline indicators (Fig. 2). Incorporating the recommendations of clinical experts, we finalize the top 12 indicators ranked in importance as the core indicators for assessing the risk of death in patients with sepsis.

**Table 1:** Baseline indicators.

Baseline indicators		
Albumin min	BUN max	PT max
ALP max	Serum chlorine max	PTT max
ALT max	Serum creatinine max	Serum sodium max
Anion gap max	INR max	Serum calcium max
AST max	lactate max	White blood cell max
Bicarbonate max	PH max	Blood glucose max
Total bilirubin max	Serum potassium max	Age

The core indicators for assessing the risk of death in patients with sepsis are shown in Table 2. Surviving patients have higher albumin, bicarbonate, and PH. In contrast, ALP, total bilirubin, serum chloride, creatinine, lactate, serum calcium, white blood cell, blood glucose, and age are higher in the deceased patients.

**Table 2:** Core indicators.

Indicators	All(mean) N = 9432	Survivors(mean) N = 7506	Decedents(mean) N = 1926
Albumin	2.950	3.077	2.454
ALP	186.180	170.030	249.118
Bicarbonate	29.258	29.830	27.026
Total bilirubin	2.781	1.944	6.042
Serum chlorine	109.988	109.778	110.807
Serum creatinine	2.790	2.578	3.619
Lactate	3.485	2.677	6.635
PH	7.434	7.435	7.431
Serum calcium	9.162	9.152	9.201
White blood cell	19.045	17.676	24.380
Blood glucose	239.672	233.829	262.447
Age	63.162	62.202	66.900

Zhang et al.^29^ believe that serum sodium has a strong relationship to in-hospital mortality in patients with sepsis; SAPS II considers serum potassium, serum sodium, and BUN to be the core indicators for assessing the risk of death in patients with sepsis; Hu et al.^12^ consider BUN to be the core indicators for assessing the risk of death in patients with sepsis. However, Fig. 2 shows that the importance of three indicators, serum sodium, serum potassium and BUN, is not as significant as previous research. Therefore, we do not consider the three indicators to be core indicators for mortality risk assessment in septic patients.

### Model comparison (RC2)

**Figure 3:**
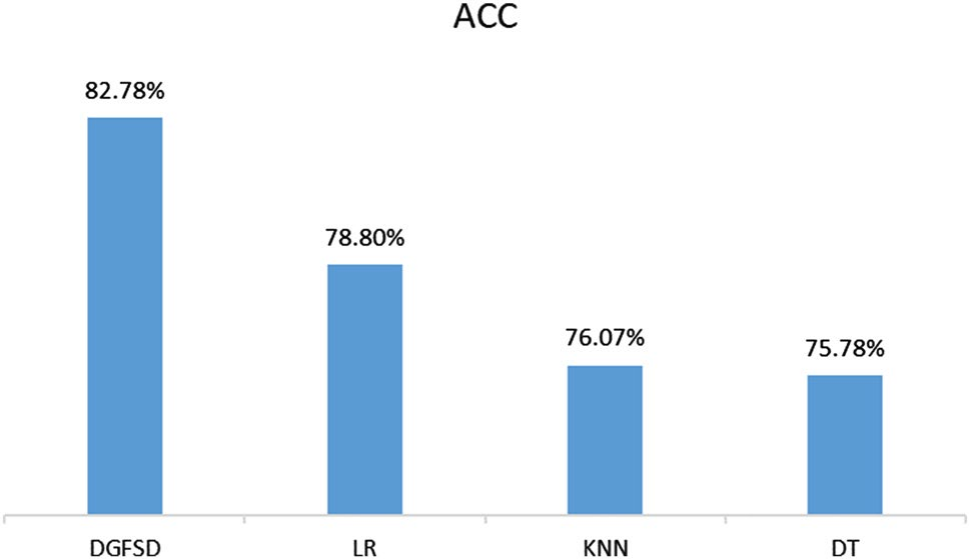
Performance evaluation of model comparison.

The experimental result of the comparison experiments of the DGSFD model with baseline models are shown in Fig. 3. The DGFSD model outperforms the baseline models. DGFSD, as a deep learning model, have an accuracy of 82.78%, which is superior to LR (78.80%), DT (75.78%) and KNN (76.07%). It shows that the DGFSD model can be used for the accurate clinical diagnosis.

### Ablation study (RC3)

**Figure 4:**
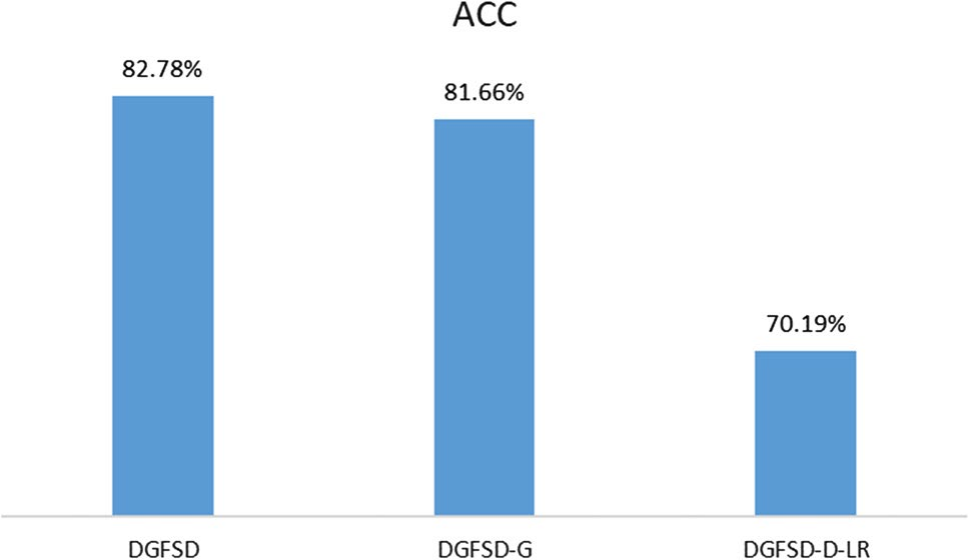
Performance evaluation of ablation study.

The results of the ablation experiments of the DGFSD model are shown in Fig. 4. The DGFSD model can not only learn individual clinical information about unassessed patients, but also obtain information about the structure of the similarity graph between diagnosed patients and patients to be assessed. As shown in Fig. 4, the DGFSD model has the most ascendant performance. DGFSD-G only learns the similarity graph structure information between septic patients, and the experimental result shows that it is not as accurate as the DGFSD model. Meanwhile, DGFSD-D-LR only obtains individual clinical information of sepsis patients, and experimental result shows that it gains the most powerless performance.

The ablation experiment shows that this multi-representation learning mode, DGFSD, can indeed improve the performance of the model in assessing the risk of death in sepsis patients.

## Conclusions and discussions

We refine the core indicators for assessing mortality risk assessment of sepsis that are more relevant to the accurate clinical diagnosis. At the same time, We incorporate graph neural networks into the task of mortality risk assessing in septic patients, and propose a deep learning-based mortality risk assessment model DGFSD.

Specifically, we extract indicators importance rankings for mortality risk assessment of septic patients by XGboost model, and then cream off core indicators for assessing the risk of death of sepsis, taking into account the recommendations of clinicians. We construct patients similarity graph and combine two deep learning modules, DNN and GCN, to build DGFSD model. The DGFSD model can not only learn individual clinical information about unassessed patients, but also obtain information about the structure of the similarity graph between diagnosed patients and patients to be assessed. Numerous experiments have shown that the accuracy of the DGFSD model is superior to state-of-the-art methods available, and can significantly improve the efficiency of clinical auxiliary diagnosis.

Compared with existing studies, our study has several strengths. Firstly, we identify core indicators for assessing the risk of death of sepsis that are more consistent with clinical application, based on machine learning model XGboost, and in conjunction with the recommendations of clinical professional. Secondly, we improve the prediction accuracy by constructing DGFSD model that can not only learn individual clinical information about unassessed patients, but also obtain information about the structure of the similarity graph between diagnosed patients and patients to be assessed.

However, our experiments still have limitations. To begin with, MIMIC-III only contains EMRs on patients in the United States and lacks EMRs in other countries. So the validity of the DGFSD model for patients in other countries needs to be further investigated. Subsequently, effectiveness of the core indicators we refined for mortality risk assessment in septic patients need to be empirically tested in the clinical setting. In addition, many unmeasured confounding factors may have an impact on the mortality of sepsis patients, such as treatment strategies. Finally, the DGFSD model is a black-box model and the interpretability of the model requires further research.

Future research will extend the DGFSD model to heterogeneous information learning models and enhance the interpretability of the model, in addition to conducting clinical validation.

### Supplementary Information

Supplementary Information.

## Data Availability

The datasets used and/or analyzed during the current study are available from the corresponding author upon reasonable request.
