# Supplementary material for: Deep learning-based prediction of in-hospital mortality for sepsis
Source: Sci Rep. 2024 Jan 3;14:372. doi: 10.1038/s41598-023-49890-9 (PMC10764335; doi:10.1038/s41598-023-49890-9)
Supplement: Supplementary file 1 — Supplementary Information. [file 41598_2023_49890_MOESM1_ESM.docx]

**Supplementary Material**

# Deep learning-based prediction of in-hospital mortality for sepsis.

Li Yong & Liu Zhenzhou^1,*^

^1^Northwest Normal University, College of Computer Science & Engineering, Lanzhou, 730070, P.R.China.

^*^corresponding.2021222257@nwnu.edu.cn

**Supplementary material cited in main text**

**Table S1:All extracted indicators in MIMIC-Ⅲ database.**

**Figure S1: Percentages of missing data for all included indicators.**

**Table S1 All extracted indicators in MIMIC-Ⅲ database.**

| serum creatinine | Laboratory indicators |
| --- | --- |
| anion gap |  |
| lactate |  |
| blood urinary nitrogen(BUN) |  |
| PH |  |
| white blood cell |  |
| bicarbonate |  |
| ionized calcium |  |
| serum calcium |  |
| serum chloride |  |
| serum sodium |  |
| serum potassium |  |
| blood glucose |  |
| international standardized ratios(INR) |  |
| prothrombin time(PT) |  |
| partial thromboplastin time(PTT) |  |
| alanine aminotransferase(ALT) |  |
| alkaline phosphatase(ALP) |  |
| aspartate aminotransferase(AST) |  |
| total bilirubin |  |
| creatine kinase MB |  |
| lactate dehydrogenase |  |
| hematocrit |  |
| albumin |  |
| age | Vital signs |
| heart rate |  |
| respiratory rate |  |
| body temperature |  |
| oxygen saturation |  |
| Glasgow Coma Scale (GCS) score |  |

**Figure S1 Percentages of missing data for all included indicators.**
